# Prognostic impact and immunotherapeutic implications of NETosis-related prognostic model in clear cell renal cell carcinoma

**DOI:** 10.1007/s00432-024-05761-y

**Published:** 2024-05-27

**Authors:** Xingjun Mao, Wen Huang, Qing Xue, Xiaolei Zhang

**Affiliations:** 1 Department of Urology, Baoying People’s Hospital, Xincheng Road, Baoying, Yangzhou, 225800 Jiangsu China; 2 Department of Good Clinical Practice Office, Nanjing First Hospital, Nanjing Medical University, ChangLe Road 68, Qinhuai District, Nanjing, Jiangsu China; 3 Department of Urology, The First Affiliated Hospital of Nanjing Medical University, Nanjing, China

**Keywords:** Necroptosis, Clear cell renal cell carcinoma, Single-cell analysis, Tumor microenvironment, SLC25A37

## Abstract

**Background:**

The ramifications of necroptosis on the prognostication of clear cell renal cell carcinoma (ccRCC) remain inadequately expounded.

**Methods:**

A prognostic model delineating the facets of necroptosis in ccRCC was constructed, employing a compendium of algorithms. External validation was effectuated using the E-MTAB-1980 dataset. The exploration of immune infiltration scores was undertaken through the exploitation of multiple algorithms. Single-cell RNA sequencing data were procured from the GSE171306 dataset. Real-time quantitative PCR (RT-qPCR) was engaged to scrutinize the differential expression of SLC25A37 across cancer and paracancer tissues, as well as diverse cell lines. Assessments of proliferative and metastatic alterations in 769-P and 786-O cells were accomplished through Cell Counting Kit-8 (CCK8) and wound healing assays.

**Results:**

The necroptosis-related signature (NRS) emerges as a discerning metric, delineating patients’ immune attributes, tumor mutation burden, immunotherapy response, and drug susceptibility. Single-cell RNA sequencing analysis unveils the marked enrichment of SLC25A37 in tumor cells. Concurrently, RT-qPCR discloses the overexpression of SLC25A37 in both ccRCC tissues and cell lines. SLC25A37 knockdown mitigates the proliferative and metastatic propensities of 769-P and 786-O cells, as evidenced by CCK8 and wound healing assays.

**Conclusion:**

The NRS assumes a pivotal role in ascertaining the prognosis, tumor mutation burden, immunotherapy response, drug susceptibility, and immune cell infiltration features of ccRCC patients. SLC25A37 emerges as a putative player in immunosuppressive microenvironments, thereby providing a prospective avenue for the design of innovative immunotherapeutic targets for ccRCC.

**Supplementary Information:**

The online version contains supplementary material available at 10.1007/s00432-024-05761-y.

## Introduction

Renal cell carcinoma (RCC) constitutes 2% of the global cancer populace, with an escalating incidence (Ljungberg et al. 2022). Various histological subtypes characterize RCC, each possessing distinctive pathological features. Predominantly, clear cell renal cell carcinoma (ccRCC), originating from proximal tubular cells of the nephron, prevails as the most prevalent subtype, comprising 75% of cases (Bray et al. 2018). Current therapeutic emphasis centers on early resection as the primary intervention for ccRCC patients (Gulati and Vaishampayan 2020). Nevertheless, nearly 30% of localized ccRCC cases experience recurrence or metastasis post-tumor resection (Barata and Rini 2017). To enhance the treatment paradigm for ccRCC patients, the identification of biomarkers prognosticating early outcomes assumes paramount importance.

Cellular demise, denoting irreversible changes encompassing metabolic arrest, structural compromise, and functional abatement, is the consequence when cells endure severe damage (Tang et al. 2019; Zhang et al. 2023a). Two classifications of cell death prevail: programmed and unprogrammed (Zhang et al. 2021; Peng et al. 2022). Programmed cell death (PCD), an orchestrated and genetically determined demise, specifically refers to protective self-termination mechanisms initiated by gene regulation when cells encounter internal and external environmental factors (Zhang et al. 2023b). Inducing PCD, notably apoptosis, stands as a widely employed therapeutic strategy in cancer treatment. Unfortunately, many cancer cells manifest resistance to PCD, perpetuating unbridled proliferation (Snyder and Oberst 2021). Death receptors, aptly named, trigger PCD in tumor cells, presenting a promising avenue for effective immunotherapy and CAR T-cell therapy (Tang et al. 2019; Wei et al. 2023).

NETosis, an inflammatory cell death mode of neutrophils, manifests through the release of Neutrophil Extracellular Traps (NETs) upon activation (Fang et al. 2022). These NETs, comprising depolymerized chromatin and intracellular granular proteins, function as a potent means for neutrophils to trap and eliminate pathogens (Guillotin et al. 2022). NETosis, distinct from apoptosis and cell necrosis, is induced by various stimuli, including bacteria, fungi, viruses, activated platelets, and chemokines. Inducers promote key NETosis proteins, Neutrophil Elastase (NE), Myeloperoxidase (MPO), and Peptide Arginine Deaminase 4 (PAD4), initiating chromatin changes and culminating in the release of antibacterial proteins into the extracellular milieu (Ronchetti et al. 2021; Hu et al. 2023). However, the intricacies of the cellular signaling pathway underlying NETosis remain incompletely elucidated.

Our investigation entails the construction and validation of a NETosis-related signature (NRS) prognosticating ccRCC patient outcomes, leveraging datasets from The Cancer Genome Atlas (TCGA), the E-MTAB-1980 cohort, and GSE22541. We meticulously scrutinized the association between our model and mutational profiles, immune infiltration, immunotherapy responses, tumor microenvironment characteristics, and drug sensitivity. Furthermore, our study delved into the clinical and immune attributes of SLC25A37, a pivotal gene in necroptosis, affirming its impact on the proliferation and migration of ccRCC cells.

## Methods

### Data acquisition and processing

Transcriptomic and clinical data for ccRCC were obtained from TCGA and the Gene Expression Omnibus (GEO) databases. Sixty-seven NETosis-related Genes (NRGs) were identified through meticulous review analysis (Qi et al. 2023; Papayannopoulos 2018; Zhang et al. 2022). The E-MTAB-1980 dataset served as an external validation dataset, and single-cell sequencing data from ccRCC patients (GSE171306) (Yu et al. 2021) were procured from the Tumor Immune Single-cell Hub 2 (TISCH2) (Han et al. 2023).

### Construction of NETosis-related signature

Univariate Cox regression analysis was initially employed to screen prognostic NRGs. The TCGA-KIRC dataset served as the foundational training set, with the E-MTAB-1980 dataset as the external validation set. Ten diverse machine learning algorithms, including Lasso, Ridge, stepwise Cox, CoxBoost, random survival forest (RSF), elastic net (Enet), partial least squares regression for Cox (plsRcox), supervised principal components (SuperPC), generalized boosted regression modeling (GBM), and survival support vector machine (survival-SVM), were meticulously integrated. This intricate process, encompassing 101 algorithm combinations within a tenfold cross-validation framework, facilitated both variable selection and model creation. Models were rigorously evaluated using C-index across training and external validation sets, with rankings based on mean C-index. The selected algorithm combinations constituted the NRS, categorizing patients into low- and high-NRS groups based on the median NRS value. Prognostic differences were assessed via Kaplan–Meier (KM) curves, and NRS predictive accuracy was evaluated using receiver operating characteristic (ROC) curves, univariate analysis, and multivariable logistic regression.

### Immunogenomic landscape analysis

Quantitative analysis of immune cell abundance in the tumor microenvironment (TME) employed various algorithms. Single-sample Gene Set Enrichment Analysis (ssGSEA) computed immune cell and function enrichment scores. The ESTIMATE algorithm assessed TME scores (ImmuneScore, StromalScore, and tumor purity) for ccRCC samples. Recognizing the significance of immune subtypes, differences in NRS expression among subtypes were evaluated. Standardized immune activity scores were derived using the Tracking Tumor Immunophenotype (TIP).

### Tumor mutational burden and drug sensitivity analysis

Somatic mutations between high and low NRS groups were analyzed using the “maftools” R package. Tumor mutational burden (TMB) expression differences were assessed, and the KM curve evaluated survival based on the mutation and NRS combination. The “pRRophetic” package gauged half maximum inhibitory concentration (IC50) of nine common ccRCC chemotherapy drugs.

### Cell culture and cell transfection

Two human ccRCC cell lines (769-P, 786-O) were purchased from the cell bank of the Chinese Academy of Sciences (Shanghai, China). All cells were cultured in RPMI 1640 medium (Thermo Fisher Scientific, Inc.) supplemented with 10% fetal bovine serum (FBS; Thermo Fisher Scientific, Inc.) at a constant temperature of 37° C in a humidified atmosphere containing 5% CO2. Small interfering RNA (siRNA) that target SLC25A37 together with the nonspecific control siRNA were obtained from Dharmacon (Shanghai, China). Transfection of plasmid and siRNA was performed with Lipo2000 following the manufacturer’s instructions.

### Cell Counting Kit-8 (CCK8) assay

Briefly, 769-P and 786-O cells after different interventions were incubated in 96-well plates (2 × 10^3^), supplemented with 200 µL culture medium and conditioned in 37° C with 5% CO2. On days 1, 2 and 3, 20 μL CCK-8 solution was added into each well, and incubation was performed for 2 h. Absorbance was measured at an optical density of 450 nm using a Microplate reader (Bio-Rad Laboratories, Inc.).

### Wound-healing assay

Cell migration was assessed by performing a wound healing assay. Briefly, 769-P and 786-O cells were transfected with SLC25A37-knockdown. Approximately 2 × 10^6^ cells were seeded into 6-well plates and cultured for 24 h. Then, a yellow plastic pipette tip was used to create a wound by scraping the cells. Cell migration was monitored under a Nicon Eclipse microscope and photographed at 100×.

### Statistical analysis

All analyses were conducted using R 4.2.2. Two-sided statistical tests were applied, considering *p* value <0.05 as statistically significant. Wilcoxon *t* test compared categorized variables among risk groups, and univariate and multivariate Cox regression analyses explored NRS prognostic significance along with clinicopathological characteristics.

## Results

### Construction and prognostic analysis of the NETosis-related signature

**Fig. 1:**
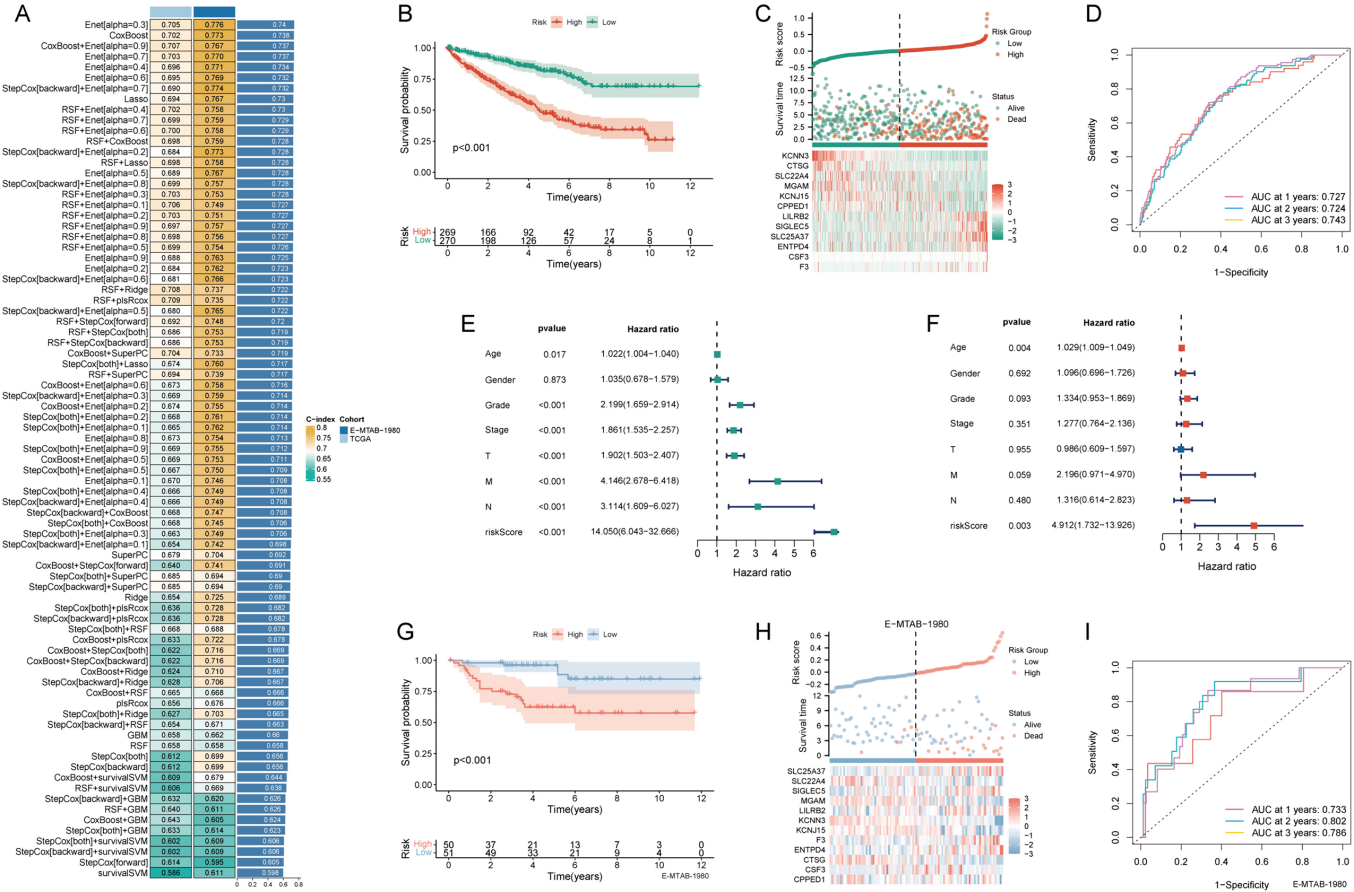
Construction and validation of the NETosis-related signature. **A** The concordance indexes (C-indexes) of 81 machine-learning algorithm combinations in the TCGA and E-MTAB-1980 cohorts; **B** Kaplan–Meier (KM) survival curve illustrating the NETosis-related signature’s prognostic utility; **C** risk curve depicting each sample reordered by the NETosis-related signature, accompanied by the scatter plot providing an overview of sample survival; **D** receiver operating characteristic (ROC) curves delineating the NETosis-related Signature’s predictive accuracy for 1, 2, and 3-year survival rates; **E**,**F** univariate and multivariate Cox regression analyses evaluating the prognostic significance of the NETosis-related signature alongside demographic and clinical parameters; **G** KM survival curve highlighting the NETosis-related signature’s prognostic value in the E-MTAB-1980 cohort; **H** risk curve of each sample reordered by the NETosis-related signature and the scatter plot of sample survival overview in the E-MTAB-1980 cohort; **I** ROC curves illustrating the NETosis-related signature’s predictive performance for 1, 2, and 3-year survival rates in the E-MTAB-1980 cohort

Univariate Cox analysis identified 34 prognostic-related NRGs out of the 67 initially considered (Table 1). For a consistent NETosis prognostic model, we meticulously employed 81 machine learning algorithms, identifying 31 prognostic genes from univariate Cox regression analysis. Within the TCGA-KIRC training set, a tenfold cross-validation framework was applied to fit 101 predictive models. The Enet [*α* = 0.3] model emerged as the optimal choice, demonstrating robust predictive performance in both the training and external validation sets. This model displayed high accuracy and clinical relevance, establishing it as a reliable and translationally meaningful predictive model (Fig. 1A). The KM survival curve depicted a negative correlation between the NRS and the chance of survival (Fig. 1B). An analysis of survival status distribution further illustrated a worse prognosis in the high NRS group (Fig. 1C). The NRS displayed area under the curve (AUC) predictive values of 0.727, 0.724, and 0.743 for the 1, 2, and 3-year survival rates, respectively (Fig. 1D). Univariate and multivariate independent prognostic analyses confirmed NRS as an independent predictor (Fig. 1E, F). External validation using the E-MTAB-19809 dataset corroborated the accuracy of NRS, evident in the KM curve, survival status distribution, and ROC curve (Fig. 1G–I).

**Table 1:** Univariate Cox analysis of 67 NETosis-related genes

ID	HR	HR.95L	HR.95H	*p* value
SLC25A37	1.092835416	1.064346554	1.122086826	4.48E−11
KCNN3	0.704029593	0.629839063	0.786959235	6.54E−10
KCNJ15	0.956225182	0.940513569	0.972199264	1.19E−07
MGAM	0.925283182	0.898128245	0.953259149	3.23E−07
DYSF	0.960678318	0.944871512	0.976749557	2.15E−06
MAPK1	0.948731375	0.927845971	0.970086901	3.59E−06
CSF3	1.240195915	1.13225585	1.358426109	3.59E−06
F3	1.013510239	1.0073042	1.019754513	1.85E−05
SLC22A4	0.908627959	0.8687394	0.950348019	2.87E−05
CPPED1	0.874378322	0.820057636	0.932297215	4.09E−05
TECPR2	0.735866083	0.633285218	0.85506321	6.22E−05
PIK3CA	0.809648912	0.729585024	0.898498926	7.05E−05
LILRB2	1.123200057	1.05968136	1.190526148	9.17E−05
VNN3	1.65621614	1.25223541	2.190524147	0.000405206
ENTPD4	1.11682061	1.04752271	1.190702847	0.000723507
CSF3R	1.047282764	1.019475252	1.075848763	0.000766155
CD93	0.989564287	0.983463553	0.995702865	0.000884808
CTSG	0.71013578	0.580071923	0.869362584	0.000912294
TLR2	1.040212662	1.01470572	1.066360778	0.001855269
CYP4F3	1.05384218	1.019269825	1.089587187	0.002059873
TLR4	0.943554259	0.909013296	0.979407719	0.002262093
G0S2	1.002636453	1.000942353	1.00433342	0.002275938
SIGLEC5	3.412334809	1.530129069	7.609834418	0.002705112
MAPK3	0.965754649	0.943874519	0.988141987	0.002880663
FPR1	1.029380143	1.008221862	1.050982446	0.00628171
BST1	0.894459158	0.820381018	0.975226348	0.011448733
MMP9	1.002223636	1.000468346	1.003982005	0.01300975
RIPK3	1.291737712	1.054886687	1.581768295	0.013249335
FCGR3B	1.0372537	1.007109034	1.068300652	0.01506842
SIGLEC14	1.095300809	1.016876613	1.179773285	0.016328977
MTOR	0.869326978	0.774807611	0.975376835	0.017102921
IL6	1.004490119	1.000639322	1.008355735	0.022248799
FPR2	1.096331428	1.011710651	1.188029995	0.024829711
CEACAM3	1.573922304	1.039642901	2.382771447	0.032056167
IL1B	1.063665046	1.003886873	1.127002814	0.036490754
ALPL	0.995127158	0.990495368	0.999780608	0.040155736
SELP	0.939104095	0.879602221	1.002631055	0.059933048
RIPK1	0.960126757	0.916747142	1.005559056	0.084535814
CLEC6A	3.324325221	0.806156335	13.70843061	0.096539602
SELPLG	1.016020815	0.996584128	1.035836581	0.106796756
IL17A	1.72E−08	2.84E−19	1044.682326	0.158180182
ATG7	0.871545291	0.716662499	1.059900853	0.168444713
ITGB2	1.006672502	0.997155723	1.016280108	0.169989643
HMGB1	0.980723512	0.952795565	1.009470071	0.186661333
AKT2	1.047939949	0.973356993	1.128237784	0.213836224
CXCR1	1.06051618	0.965597778	1.164765074	0.219377393
TLR8	1.057985569	0.964972967	1.159963544	0.229926586
CRISPLD2	1.010686307	0.993186643	1.02849431	0.232950615
CREB5	0.96358327	0.903405763	1.027769311	0.259542038
CXCR2	1.068452183	0.951932921	1.199233729	0.261082792
HPSE	0.939270785	0.82600484	1.068068326	0.339286249
TNFRSF10C	0.971890134	0.90548966	1.043159822	0.429712331
ELANE	0.930414122	0.751113593	1.152516006	0.509028194
S100A12	1.011530269	0.976862067	1.047428822	0.519376749
DNASE1	0.983753903	0.931964038	1.038421765	0.552774098
ITGAM	1.00628003	0.985682836	1.027307631	0.55297763
CYBB	1.002496114	0.994242765	1.010817976	0.554481938
PTAFR	0.99013358	0.952843506	1.028883022	0.612693353
IRAK4	0.976786701	0.890654997	1.07124786	0.618005706
MPO	0.932421267	0.670378886	1.296892606	0.677667173
TNF	0.956961467	0.777213842	1.178279645	0.678552649
MME	0.997907643	0.988032279	1.007881712	0.679768308
FCAR	1.052542903	0.803325474	1.379075602	0.710301077
AKT1	0.988586559	0.928922842	1.052082411	0.717785638
TLR7	0.988696896	0.925859444	1.055799082	0.734388677
PDE4B	1.005700727	0.937400197	1.078977747	0.874127085
PADI4	1.000479534	0.572613605	1.748053645	0.998656459

### Immunoinfiltration characteristics of NETosis-related signature

**Fig. 2:**
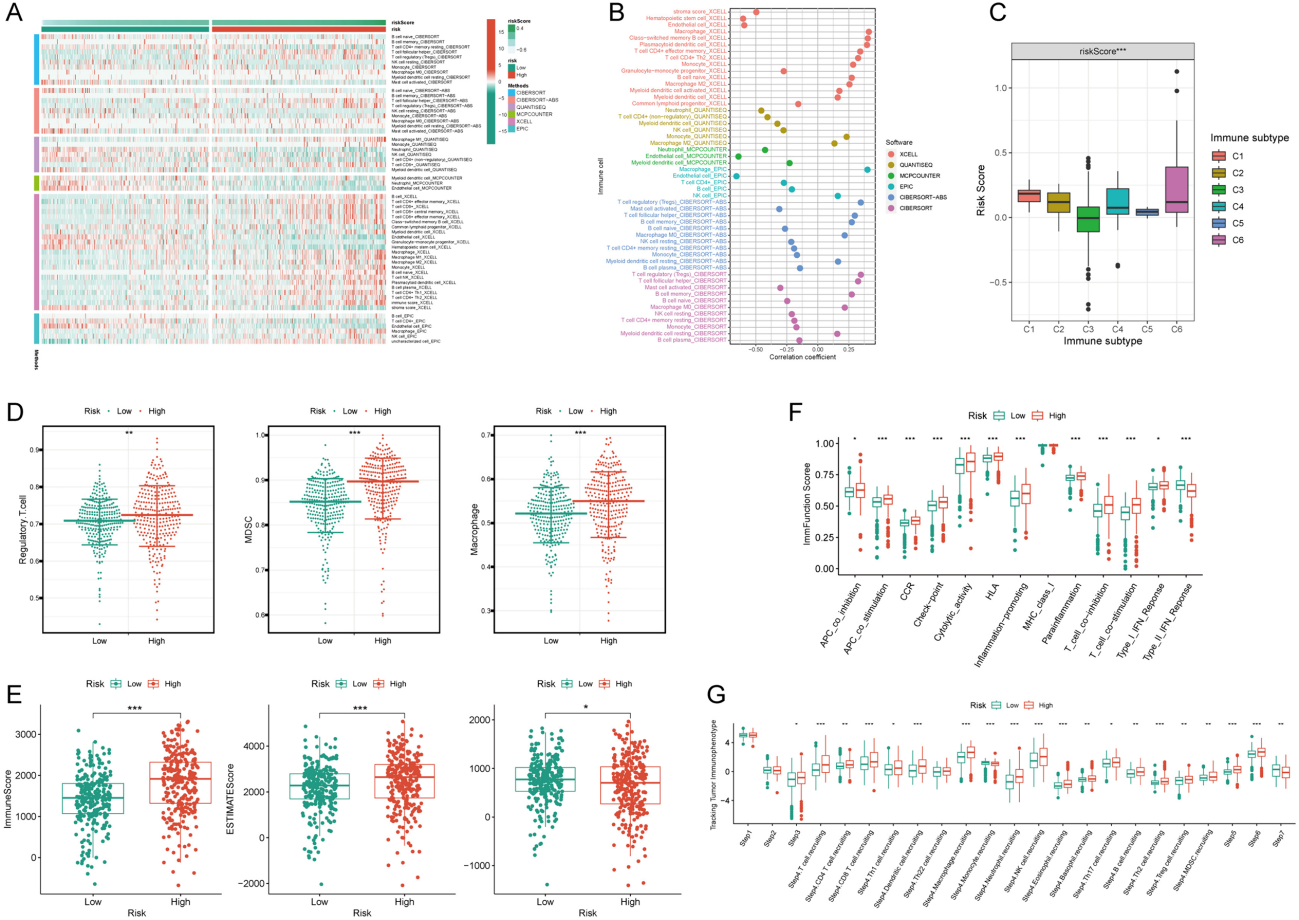
Immune infiltration characteristics of the NETosis-related signature. **A** Distribution of immune cells in high and low NETosis-related signature groups across multiple algorithms; **B** correlation analysis depicting the relationship between immune cells and the NETosis-related signature across multiple algorithms; **C** disparities in NETosis-related signature among different immune subtypes; **D** distinct expression patterns of immunosuppressive cells between high and low NETosis-related signature groups; **E** variances in tumor microenvironment scores between high and low NETosis-related signature groups; **F** contrasts in immune function scores between high and low NETosis-related signature groups; **G** differential expression patterns of the NETosis-related signature across various tracking tumor immunophenotypes. Asterisks denote statistical significance (* *p* < 0.05, ** *p* < 0.01, *** *p* < 0.001)

A heatmap vividly displayed immune cell expression across various algorithms in NRS groups (Fig. 2A). NRS exhibited positive correlations with macrophage and Regulatory T cell (Treg) cells across algorithms (Fig. 2B). The expression of NRS varied significantly among different tumor immune subtypes, with the lowest expression in C3 and the highest in C6 (Fig. 2C). C3 is immunoinflammatory and is generally associated with a better immune response and better prognosis. C5 is immune escape, in which tumor cells evade immune surveillance through various mechanisms and are associated with poor prognosis. The high NRS category manifested heightened expression of immunosuppressive cells, including Treg cells, Myeloid Derived Suppressor Cell (MDSC), and Macrophages (Fig. 2D). Additionally, StromalScore, ImmuneScore, and ESTIMATEScore were markedly upregulated in the high NRS group (Fig. 2E). The high NRS group displayed significant overexpression of immunofunctional pathways like Checkpoint, HLA, inflammation promotion, and Parainflammation (Fig. 2F). In-depth analysis utilizing the TIP database revealed significantly higher immune cell frequency in the high NRS group (Fig. 2G), suggesting a potential correlation between NRS and immune cell activity in ccRCC.

### Tumor mutation burden characteristics and drug sensitivity of NETosis-related signature

**Fig. 3:**
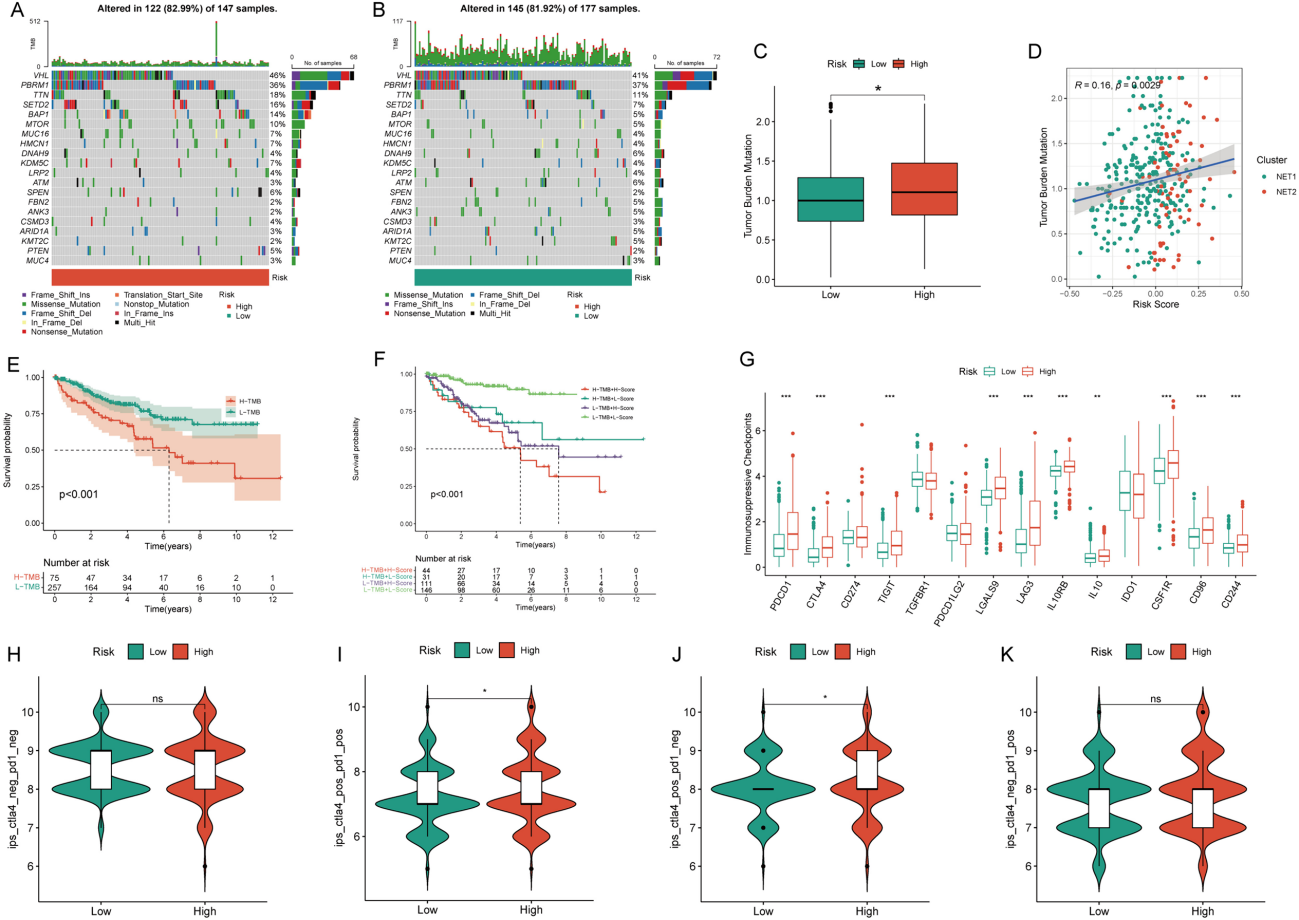
Tumor mutation burden and immunotherapy characteristics of the NETosis-related signature. **A**,**B** Waterfall chart visualizing the mutation frequency of the top 20 genes in the high and low NETosis-related signature groups; **C** disparities in tumor mutation burden (TMB) between high and low NETosis-related signature groups; **D** correlation analysis assessing the relationship between the NETosis-related signature and TMB; **E** KM survival curve illustrating prognostic differences between high and low TMB groups; **F** KM survival curve evaluating the combined prognostic impact of the NETosis-related signature and TMB; **G** contrasts in the expression of immunosuppressive checkpoints between high and low NETosis-related signature groups; **H**–**K** differences in immunotherapy response between high and low NETosis-related signature groups

The high NRS group exhibited a mutation rate of 82.99%, contrasting with the 81.92% rate observed in the low NRS group (Fig. 3A, B). Notably, the high NRS group displayed significantly elevated TMB levels (Fig. 3C), with a clear positive correlation between NRS and TMB (Fig. 3D). The KM survival curve demonstrated a connection between high TMB and unfavorable prognosis (Fig. 3E). Survival analysis combining TMB and NRS affirmed that the high-NRS and high-TMB subgroup displayed the poorest prognosis (Fig. 3F). TMB was also associated with better immunotherapy outcomes, with most immunosuppressive checkpoints significantly overexpressed in the high-risk group (Fig. 3G). Immunotherapy data from the TCIA database indicated that high-risk patients were more likely to respond positively to immunotherapy, particularly with CTLA4 and CTLA4+PD-1 combinations, thus demonstrating a better prognosis (Fig. 3H–K). Evaluating nine primary chemotherapeutic drugs revealed higher IC50 values for Sorafenib, Temsirolimus, Sunitinib, Metformin, Mitomycin C, Lenalidomide, Bosutinib, and Gefitinib in the low NRS group, except for Axitinib, where the low NRS group exhibited elevated IC50 values (Figure S1A–I).

### Expression and prognostic characteristics of NETosis-related genes

**Fig. 4:**
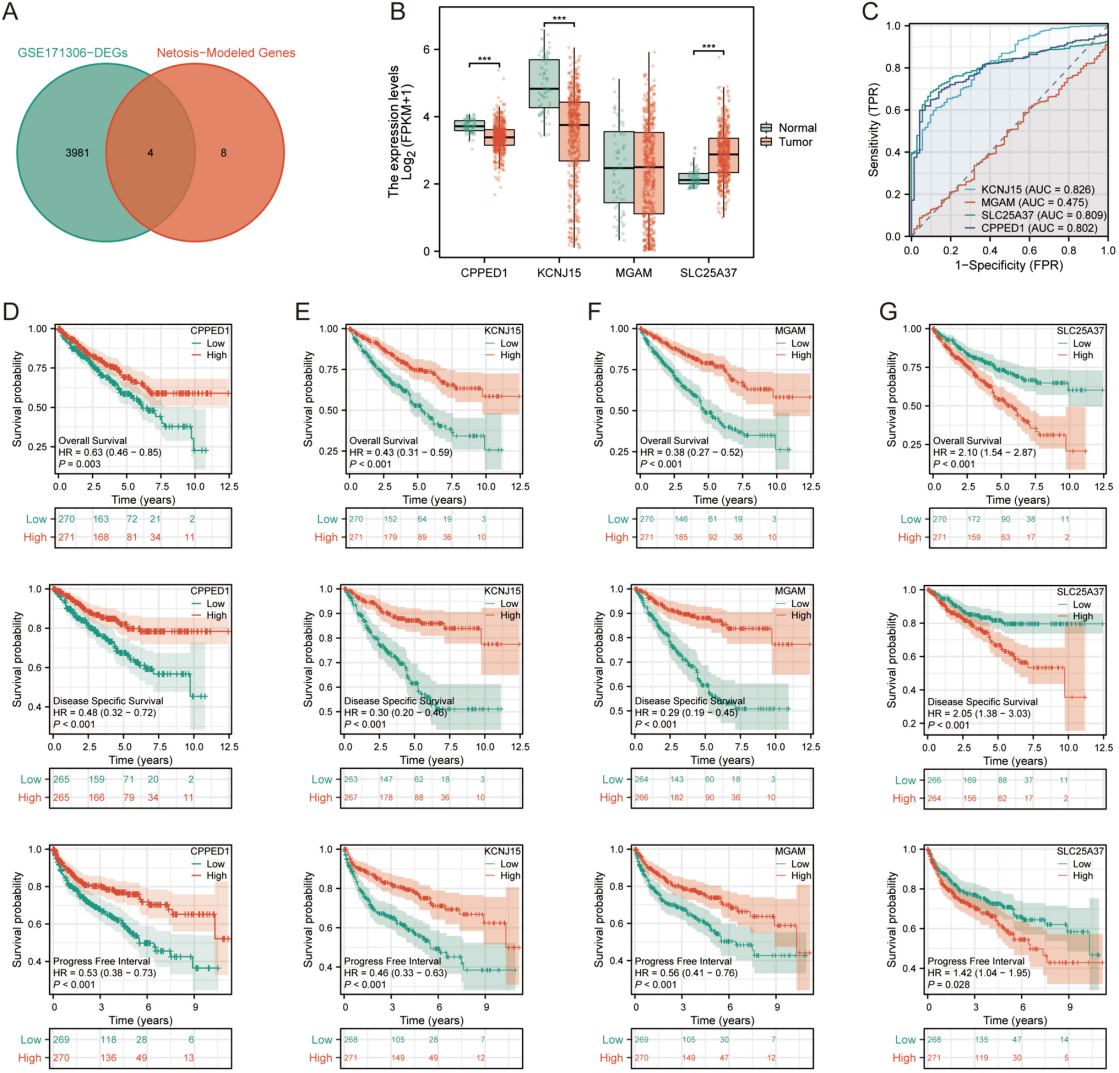
Prognostic characteristics of major NETosis genes. **A** Intersection of differentially expressed genes from GSE171306 and NETosis-related genes; **B** disparities in expression of intersection genes between ccRCC and paracancer tissues; **C** diagnostic and predictive value assessment of intersection genes; **D**–**G** KM survival analyses for overall survival (OS), progress-free interval (PFI), and disease-specific survival (DSS) of intersection genes

We obtained data on differentially expressed genes in GSE171306 from the TISCH2 database. Subsequently, we filtered out 3985 genes showing differential expression between tumor cells and other cells based on an adjusted *p* value <0.001 criterion. We then intersected these genes with those used to construct the NETosis prognostic model. Ultimately, four NRGs (CPPED1, KCNJ15, MGAM, SLC25A37) with significant differential expression were identified (Fig. 4A). In the TCGA dataset, SLC25A37 was significantly upregulated in tumor tissues, while CPPED1 and KCNJ15 were significantly upregulated in adjacent non-tumor tissues. MGAM exhibited no statistically significant difference between cancerous and adjacent non-tumor tissues (Fig. 4B). AUC values indicated that CPPED1, KCNJ15, and SLC25A37 had superior predictive capabilities (Fig. 4C). High expression of CPPED1, KCNJ15, MGAM was associated with significantly longer Overall Survival (OS), Progress Free Interval (PFI), and Disease Specific Survival (DSS) (Fig. 4D–F). Conversely, low SLC25A37 expression correlated with significantly longer OS, DSS, and PFI (Fig. 4G).

### Expression landscape of NETosis-related genes in multiple cells

**Fig. 5:**
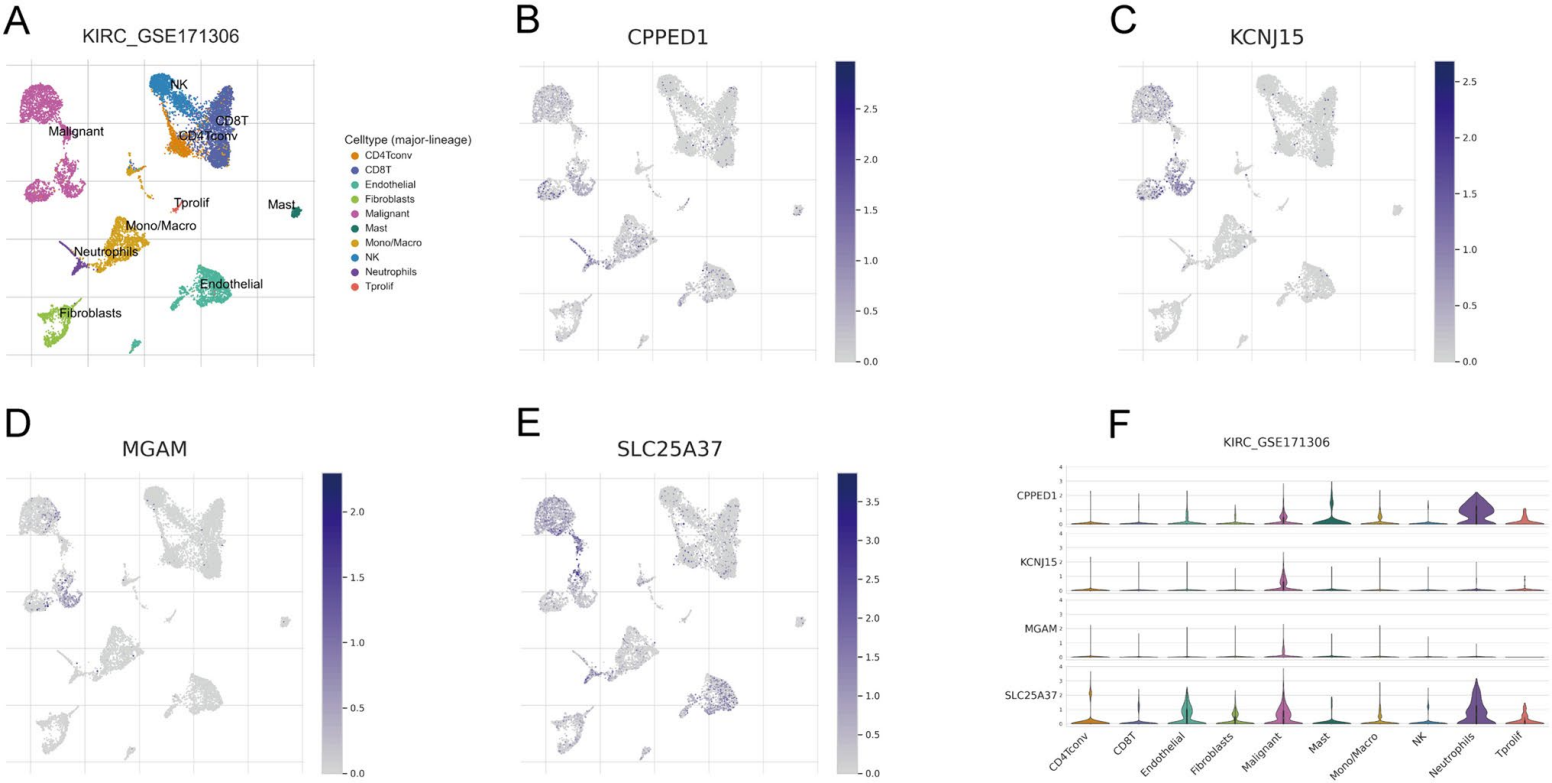
Expression profile of intersection genes based on single-cell sequencing analysis. **A** Clustering of ten cell types in GSE171306; **B**–**E** expression distribution of intersection genes in each cell; **F** specific expression patterns of intersection genes in ten cell types

GSE171306 was meticulously annotated and classified into ten distinct cell types, encompassing immune cells, malignant cells, and stromal cell subsets, as illustrated in Fig. 5A. This comprehensive categorization provided a detailed landscape of the cellular composition within the dataset. Figure 5B–E vividly portray the expression and distribution patterns of the four NRGs across different cell types. Notably, the expression of SLC25A37 exhibited its peak in malignant cells, suggesting a potential significance of this gene in the context of malignancy. Figure 5F presented the expression profiles of the four NRGs across various cell types. The results underscored that SLC25A37 displayed the highest expression levels in malignant cells and neutrophils, shedding light on its potential role in these specific cell types within the ccRCC microenvironment.

### Clinical and prognostic features of SLC25A37

**Fig. 6:**
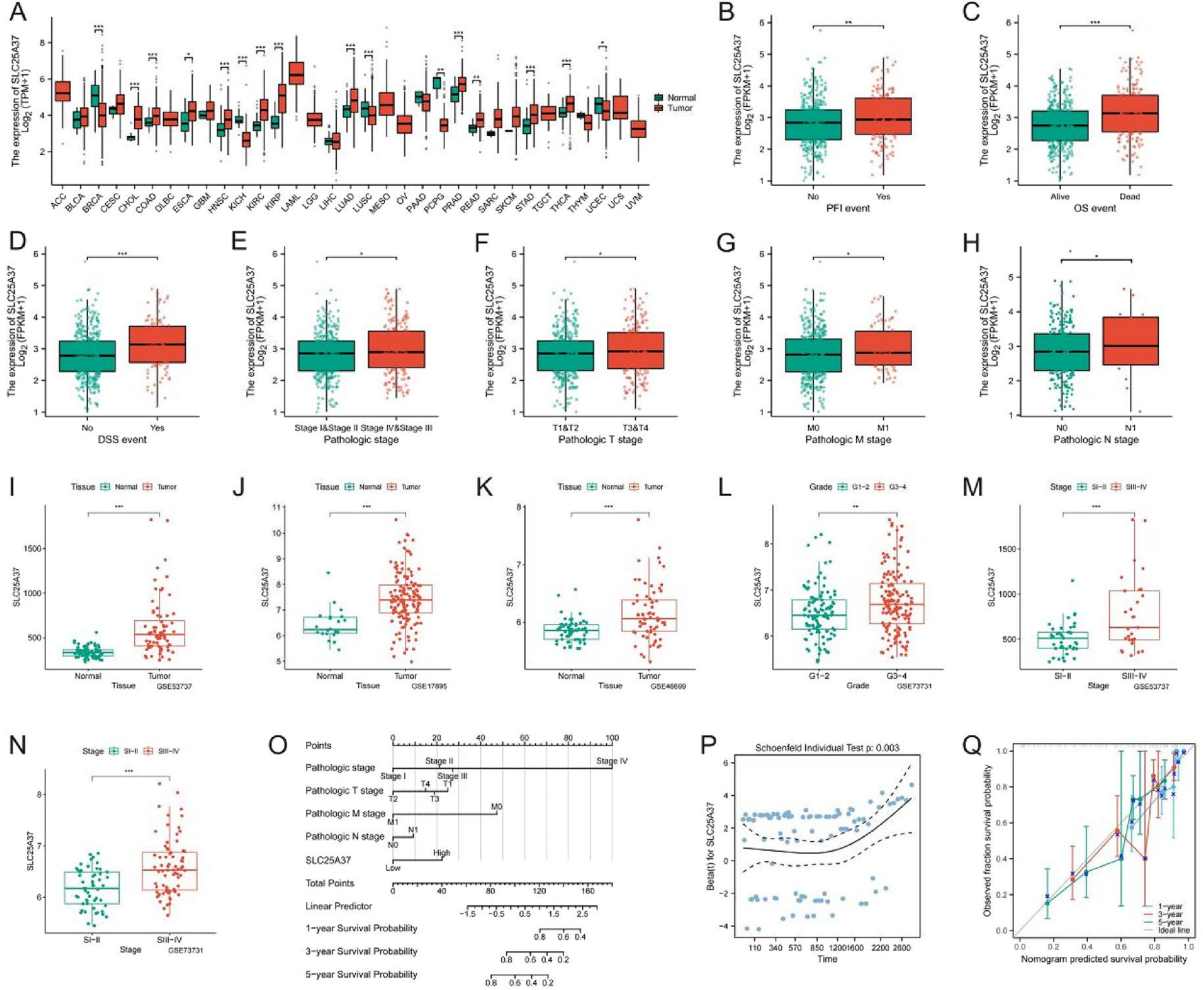
Correlation of SLC25A37 with clinicopathological characteristics in ccRCC. **A** Differential expression of SLC25A37 in 33 tumors and adjacent tissues; **B**–**H** contrasts in SLC25A37 expression profiles among clinicopathological variables (**B** PFI; **C** OS; **D** DSS; **E** stage; **F** T stage; **G** M stage; **H** N stage); **I**–**N** variances in SLC25A37 expression across different clinicopathological stages in the GEO validation datasets; **O** nomogram predicting 1-, 3-, and 5-year OS of ccRCC patients; **P** prognostic risk hypothesis diagram for SLC25A37; **Q** prognostic calibration curve of SLC25A37

Given the substantial and significant overexpression of SLC25A37 in tumors, coupled with its favorable prognostic characteristics, we chose to focus our further analysis on this gene. The expression of SLC25A37 in 33 tumors is illustrated in Fig. 6A. Subsequent examination of TCGA data revealed a noteworthy upregulation of SLC25A37 in patients who experienced mortality, those at advanced stages, and those with metastasis (Fig. 6B–H). Our validation across diverse GEO datasets consistently confirmed a significant overexpression of SLC25A37 in both tumors and advanced stages (Fig. 6I–N). Additionally, based on the expression profile of SLC25A37, we developed a Normo diagram, prognostic risk hypothesis diagram, and prognostic calibration curve, demonstrating the gene’s robust prognostic accuracy (Fig. 6O–Q).

### Identification of immune infiltration characteristics of SLC25A37

**Fig. 7:**
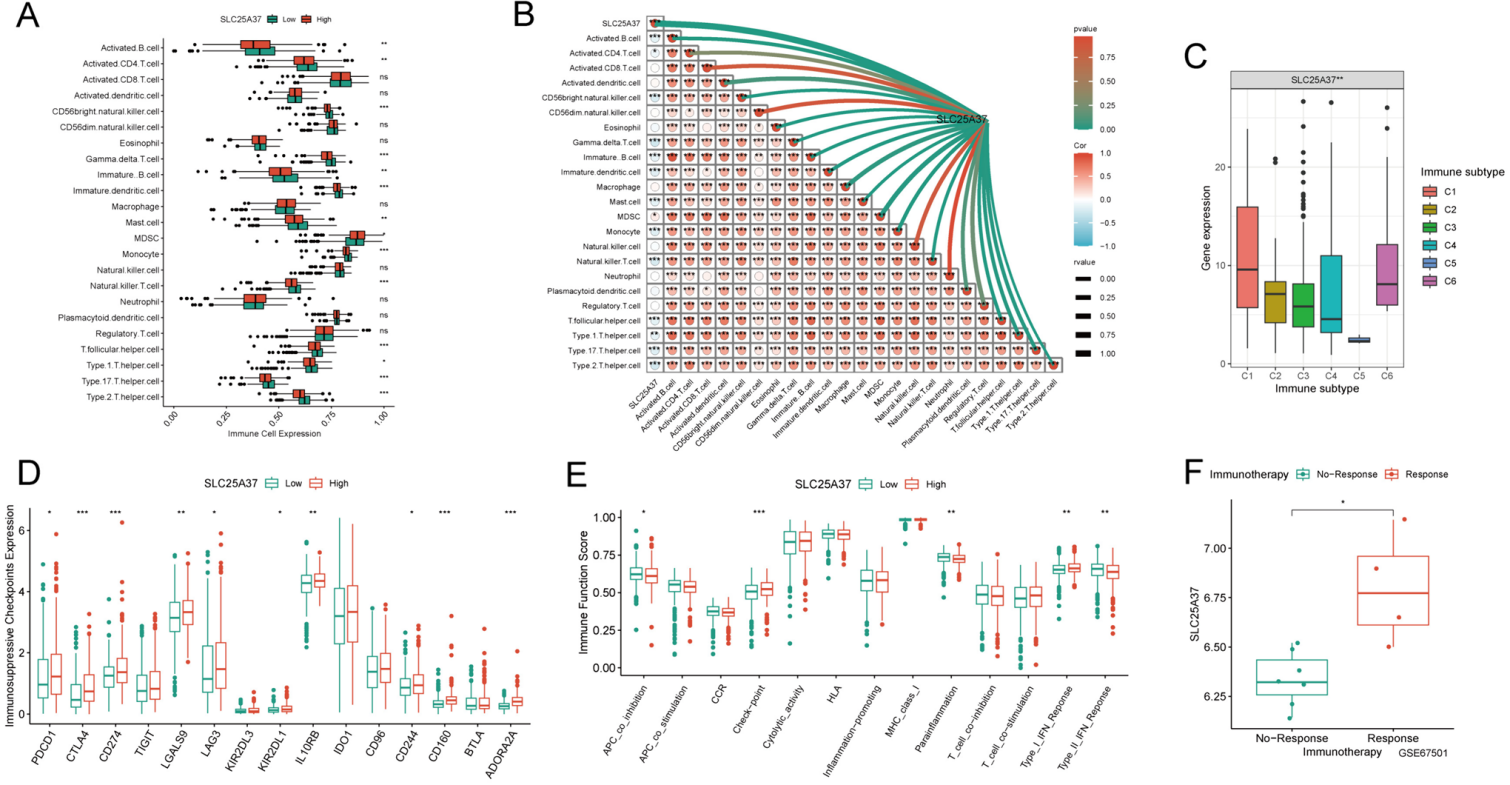
Identification of immune infiltration characteristics of SLC25A37 in ccRCC. **A** Disparities in the expression of immune cells between high and low SLC25A37 groups; **B** correlation analysis assessing the relationship between immune cells and SLC25A37; **C** contrasts in SLC25A37 expression among different immune subtypes; **D** differences in the expression of immunosuppressive checkpoints between high and low SLC25A37 groups; **E** variances in immune function scores between high and low SLC25A37 groups; **F** expression differences of SLC25A37 among various immunotherapy outcomes

Subsequently, we conducted an in-depth analysis of the immune profile associated with SLC25A37. Remarkably, the majority of immune cells exhibited significant underexpression in the high-SLC25A37 group, with a strong and consistent negative correlation observed between SLC25A37 and most immune cells (Fig. 7A, B). Concurrently, our investigation revealed a significant overexpression of SLC25A37 in immunosubtype 6, aligning with a prognostically unfavorable outcome (Fig. 7C). Further scrutiny uncovered a noteworthy upregulation of immunosuppressive checkpoints within the high SLC25A37 group (Fig. 7D). Evaluation of the immune function score indicated a significantly higher checkpoint score in the high SLC25A37 group compared to the low SLC25A37 group (Fig. 7E). Given the pivotal role of immune checkpoints in determining responses to immunotherapy, our analysis of the GSE67501 dataset disclosed a significant elevation of SLC25A37 expression in the immunotherapy response group (Fig. 7F). Consequently, SLC25A37 emerges as a promising target for immunotherapy, offering novel perspectives and avenues for advancing immunotherapeutic strategies.

### SLC25A37-knockdown suppressed proliferation and migration in ccRCC cells

**Fig. 8:**
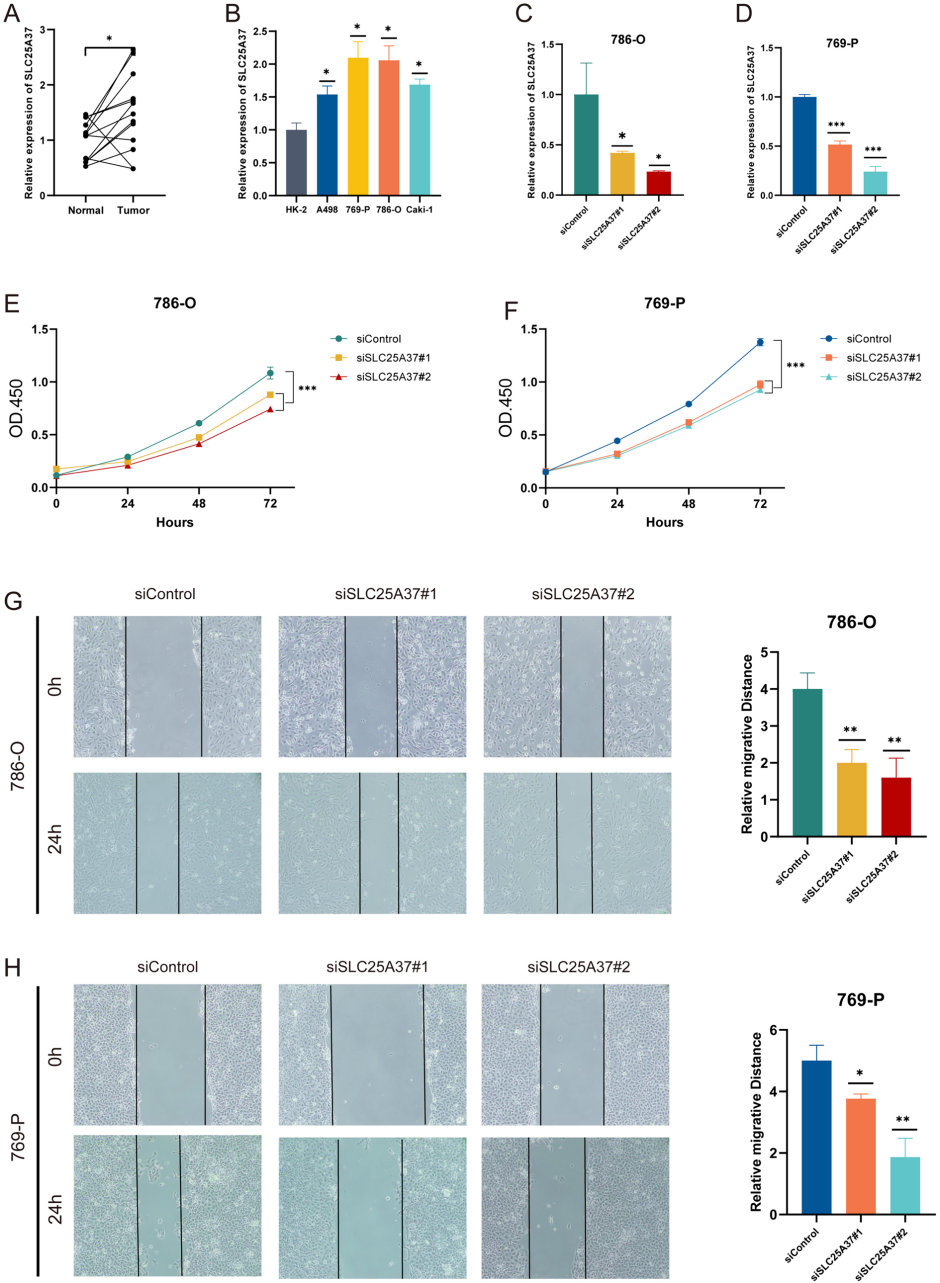
SLC25A37 knockdown suppresses proliferation and migration in ccRCC cells. **A**,**B** RT-qPCR analysis of SLC25A37 expression in ccRCC tissues and cell lines; **C**,**D** detection of SLC25A37 knockdown in 786-O and 769-P cells by RT-qPCR; **E**,**F** inhibition of proliferation in 769-P and 786-O cells following SLC25A37 knockdown; **G**,**H** suppression of migration in 769-P and 786-O cells upon SLC25A37 knockdown

RT-qPCR validated the significantly higher expression of SLC25A37 in ccRCC tissues (Fig. 8A). Moreover, compared with normal cell lines, SLC25A37 was significantly over-expressed in ccRCC cell lines, especially in 786-O and 769-P cell lines (Fig. 8B). Transfection with SLC25A37-siRNA effectively knocked down SLC25A37, confirmed by RT-qPCR (Fig. 8C, D). CCK8 assay demonstrated reduced proliferation in both 769-P and 786-O cells post-SLC25A37 knockdown (Fig. 8E, F). Wound-healing assay indicated decreased migration in SLC25A37-knockdown 769-P and 786-O cells compared to control (Fig. 8G, H).

## Discussion

NETosis, a form of programmed cell death initiated by NETs, represents a distinctive neutrophil death mode (Li et al. 2023). Simply put, the process by which NETs are formed is called NETosis (Adrover et al. 2023). NETs, composed of DNA and released contents from activated neutrophils, serve as a crucial mechanism for trapping and eliminating bacteria and fungi (Kaltenmeier et al. 2021). Tumor cells strategically release cytokines and chemokines to recruit neutrophils to the TME (Andrea et al. 2021). Within the TME, neutrophils are stimulated to induce NETs production, forming a protective network around cancer cells. This network acts as a physical barrier, shielding tumor cells from cytotoxic T lymphocytes and Natural Killer (NK) cells (Vorobjeva and Chernyak 2020). Recent research highlights the role of NETs in promoting tumor development, metastasis, and chemotherapy resistance across various cancers, including gastric cancer, liver cancer, melanoma, and more (Zhou et al. 2023; Yin et al. 2023; Weide et al. 2023). Targeting NETs emerges as a promising avenue for novel tumor treatments.

In our study, we employed diverse algorithms to meticulously screen and construct a NETosis-related prognostic model, demonstrating high accuracy. This model exhibited superior prognostic prediction capabilities in the validation set, presenting a valuable tool for assessing the prognosis of ccRCC patients. The TME plays a pivotal role in tumor proliferation, metastasis, and anti-cancer immune regulation. In our investigation, the high NRS group exhibited elevated tumor microenvironment scores and increased immunosuppressive cell infiltration. These findings correlated with a poorer prognosis, emphasizing the significance of invasive immune cells as therapeutic targets (Pitt et al. 2016). Targeted therapy and immune checkpoint blocking therapy are integral components of ccRCC treatment (Ravi et al. 2020). Notably, our study revealed that patients with high NRS responded more favorably to CTLA4 and CTLA4 + PD-L1 treatments, suggesting a potential stratification strategy for individualized treatment plans. At the same time, many studies have found that Netois-related prognostic models play a crucial role in other tumors. For example, Feng et al. developed a NETs scoring system to quantify NETs patterns in colon cancer patients, helping to provide an ideal strategy for individualized treatment (Feng et al. 2023). Tian et al. found that Netosis prognostic model could predict the prognosis of gastric cancer patients more accurately (Xiang et al. 2023). The difference of our study is that we first used a combination of multiple algorithms to build a prognostic model, which is relatively comprehensive. In addition, we screened out the key gene SLC25A37 and further verified its effect on ccRCC cell proliferation and migration through experiments.

Recent insights underscore RCC, particularly ccRCC, as a metabolic disease characterized by significant alterations in cellular metabolism (Meo et al. 2023, 2022; Lucarelli et al. 2019; Marco et al. 2023). These include a reprogrammed glycolytic flux, impaired mitochondrial bioenergetics, and dysfunctional lipid metabolism (Lucarelli et al. 2018, 2022; Bombelli et al. 2020; Bianchi et al. 2017), reflecting a metabolic adaptation essential for tumor survival and growth (Bianchi et al. 2017; Lucarelli et al. 2015). Moreover, the involvement of NET-osis in the regulation of cell metabolism introduces an additional layer of complexity. NET-osis, a unique form of NET formation, has been implicated in various cancers as a modulator of the tumor microenvironment, influencing both metabolic reprogramming and immune evasion mechanisms. In this context, our findings reveal a pivotal role of NET-osis in ccRCC progression. The NRS developed in our study not only correlates with immune infiltration but also with the metabolic reprogramming observed in ccRCC, suggesting that targeting metabolic pathways and NET-osis could be a promising therapeutic strategy. These insights align with recent studies which highlight the potential of targeting metabolic alterations and NET-osis in cancer therapy. As such, integrating the metabolic perspective into the management of RCC could lead to more precise and effective therapeutic interventions.

RCC is characterized by substantial immune infiltration, which significantly impacts the tumor’s biology and response to therapy (Vuong et al. 2019; Tamma et al. 2019; Gigante et al. 2016). The metabolic pathways active in RCC are crucial for supporting not just cellular energy demands but also for driving angiogenesis and sustaining inflammatory conditions within the TME (Netti et al. 2020; Lucarelli et al. 2017). These metabolic shifts create a milieu that can either suppress or stimulate immune activity, thereby affecting disease progression and therapeutic outcomes (Lasorsa et al. 2023a, b). NET-osis, a process involving the formation of NETs, further influences these interactions by altering how immune cells respond within the TME (Ghini et al. 2020; Lucarelli et al. 2023; Lasorsa et al. 2023c). NETs can trap and kill pathogens, but in cancer, they also modulate immune responses and promote a chronic inflammatory state, which supports tumor growth and metastasis. Understanding the dual roles of NET-osis in immune regulation and inflammation highlights its potential as a therapeutic target in RCC. By diving deeper into how metabolic reprogramming and NET-osis influence the immune landscape in RCC, we can better understand the mechanisms underlying tumor aggressiveness and resistance to treatments. This knowledge is crucial for developing targeted therapies that can disrupt these processes and improve patient outcomes in RCC.

The solute carrier (SLC) family of transporters assumes a pivotal role in orchestrating the flux of metabolites into and out of mitochondria (Lin et al. 2015). These mitochondrial membrane transport proteins are strategically positioned within the inner membrane of mitochondria, acting as conveyors for metabolic substrates (Lewis et al. 2021). In the realm of human biology, the SLC25 encodes 53 transporters. Perturbations in the genetic sequence or aberrant expression of SLC25 instigate disturbances in metabolism, giving rise to both neoplastic and non-neoplastic maladies. A corpus of scientific literature underscores the up-regulation of SLC25A8 in colon cancer, establishing a correlation with tumor differentiation (Horimoto et al. 2004). Noteworthy research by Kuai reveals that SLC25A14, through a feedback mechanism for mitochondrial dysfunction, exerts inhibitory control over the escalation of H2O2 products in colon cancer (Kuai et al. 2010). Compelling evidence supports the consistent downregulation of SLC25A37 in individuals afflicted with major depressive disorder (MDD), heralding its potential utility as a biomarker for MDD diagnosis and a propitious target for subsequent therapeutic and diagnostic interventions (Huo et al. 2016). In the purview of our investigation, we discerned a significant overexpression of SLC25A37 in ccRCC, concomitant with an ominous prognosis. Furthermore, a discernible negative correlation manifested between SLC25A37 and immune cells, intimating its potential role in shaping an immunosuppressive microenvironment. Concurrently, our findings underscored the noteworthy capacity of SLC25A37 silencing to markedly impede the proliferation and migration of ccRCC cells.

Despite these promising findings, our study acknowledges its limitations, primarily relying on publicly available data for retrospective analysis. Larger sample sizes and prospective studies are warranted to evaluate the clinical utility of our findings in ccRCC patients. Additionally, further in vivo and in vitro experiments are essential to elucidate the regulatory role of SLC25A37 in NETosis and its impact on ccRCC progression.

## Conclusion

In summary, we have successfully formulated a NETosis-related prognostic model in ccRCC. This model stands poised as a robust tool for forecasting both prognosis and immune responsiveness. Our investigation has implicated SLC25A37 in fostering the proliferation and migration of tumor cells in ccRCC. Moreover, our findings suggest a potential role for SLC25A37 in orchestrating the assembly of an immunosuppressive TME, thereby presenting itself as a plausible therapeutic target for the management of ccRCC.

## Supplementary Information

Below is the link to the electronic supplementary material.

Supplementary file1 (DOCX 123 KB)

## Data Availability

The data that support the findings of this study are openly available in TCGA (https://www.cancer.gov/ccg/research/genome-sequencing/tcga/) and GEO (https://www.ncbi.nlm.nih.gov/geo/) datasets.

## References

[CR1] Adrover JM, McDowell SAC, He XY, Quail DF, Egeblad M (2023) NETworking with cancer: the bidirectional interplay between cancer and neutrophil extracellular traps. Cancer Cell 41:505–526 36827980 10.1016/j.ccell.2023.02.001 PMC10280682

[CR2] Barata PC, Rini BI (2017) Treatment of renal cell carcinoma: current status and future directions. CA Cancer J Clin 67:507–524 28961310 10.3322/caac.21411

[CR3] Bianchi C, Meregalli C, Bombelli S, Di Stefano V, Salerno F, Torsello B, De Marco S, Bovo G, Cifola I, Mangano E, Battaglia C, Strada G, Lucarelli G, Weiss RH, Perego RA (2017) The glucose and lipid metabolism reprogramming is grade-dependent in clear cell renal cell carcinoma primary cultures and is targetable to modulate cell viability and proliferation. Oncotarget 8:113502–113515 29371925 10.18632/oncotarget.23056 PMC5768342

[CR4] Bombelli S, Torsello B, De Marco S, Lucarelli G, Cifola I, Grasselli C, Strada G, Bovo G, Perego RA, Bianchi C (2020) 36-kDa annexin A3 isoform negatively modulates lipid storage in clear cell renal cell carcinoma cells. Am J Pathol 190:2317–2326 32861643 10.1016/j.ajpath.2020.08.008

[CR5] Bray F, Ferlay J, Soerjomataram I, Siegel RL, Torre LA, Jemal A (2018) Global cancer statistics 2018: GLOBOCAN estimates of incidence and mortality worldwide for 36 cancers in 185 countries. CA Cancer J Clin 68:394–424 30207593 10.3322/caac.21492

[CR6] de Andrea CE, Ochoa MC, Villalba-Esparza M, Teijeira Á, Schalper KA, Abengozar-Muela M, Eguren-Santamaría I, Sainz C, Sánchez-Gregorio S, Garasa S, Ariz M, Ortiz-de-Solorzano C, Rodriguez-Ruiz ME, Perez-Gracia JL, Lozano MD, Echeveste JI, Sanmamed MF, Melero I (2021) Heterogenous presence of neutrophil extracellular traps in human solid tumours is partially dependent on IL-8. J Pathol 255:190–201 34184758 10.1002/path.5753

[CR7] De Marco S, Torsello B, Minutiello E, Morabito I, Grasselli C, Bombelli S, Zucchini N, Lucarelli G, Strada G, Perego RA, Bianchi C (2023) The cross-talk between Abl2 tyrosine kinase and TGFβ1 signalling modulates the invasion of clear cell renal cell carcinoma cells. FEBS Lett 597:1098–1113 36310399 10.1002/1873-3468.14531

[CR8] di Meo NA, Lasorsa F, Rutigliano M, Loizzo D, Ferro M, Stella A, Bizzoca C, Vincenti L, Pandolfo SD, Autorino R, Crocetto F, Montanari E, Spilotros M, Battaglia M, Ditonno P, Lucarelli G (2022) Renal cell carcinoma as a metabolic disease: an update on main pathways, potential biomarkers, and therapeutic targets. Int J Mol Sci 23:14360 36430837 10.3390/ijms232214360 PMC9698586

[CR9] di Meo NA, Lasorsa F, Rutigliano M, Milella M, Ferro M, Battaglia M, Ditonno P, Lucarelli G (2023) The dark side of lipid metabolism in prostate and renal carcinoma: novel insights into molecular diagnostic and biomarker discovery. Expert Rev Mol Diagn 23:297–313 36960789 10.1080/14737159.2023.2195553

[CR10] Fang Q, Stehr AM, Naschberger E, Knopf J, Herrmann M, Stürzl M (2022) No NETs no TIME: crosstalk between neutrophil extracellular traps and the tumor immune microenvironment. Front Immunol 13:1075260 36618417 10.3389/fimmu.2022.1075260 PMC9816414

[CR11] Feng C, Li Y, Tai Y, Zhang W, Wang H, Lian S, Jin-Si-Han EE, Liu Y, Li X, Chen Q, He M, Lu Z (2023) A neutrophil extracellular traps-related classification predicts prognosis and response to immunotherapy in colon cancer. Sci Rep 13:19297 37935721 10.1038/s41598-023-45558-6 PMC10630512

[CR12] Ghini V, Laera L, Fantechi B, Monte FD, Benelli M, McCartney A, Leonardo T, Luchinat C, Pozzessere D (2020) Metabolomics to assess response to immune checkpoint inhibitors in patients with non-small-cell lung cancer. Cancers 12:3574 33265926 10.3390/cancers12123574 PMC7760033

[CR13] Gigante M, Pontrelli P, Herr W, Gigante M, D’Avenia M, Zaza G, Cavalcanti E, Accetturo M, Lucarelli G, Carrieri G, Battaglia M, Storkus WJ, Gesualdo L, Ranieri E (2016) miR-29b and miR-198 overexpression in CD8+ T cells of renal cell carcinoma patients down-modulates JAK3 and MCL-1 leading to immune dysfunction. J Transl Med 14:84 27063186 10.1186/s12967-016-0841-9 PMC4827202

[CR14] Guillotin F, Fortier M, Portes M, Demattei C, Mousty E, Nouvellon E, Mercier E, Chea M, Letouzey V, Gris JC, Bouvier S (2022) Vital NETosis vs. suicidal NETosis during normal pregnancy and preeclampsia. Front Cell Dev Biol 10:1099038 36684420 10.3389/fcell.2022.1099038 PMC9849884

[CR15] Gulati S, Vaishampayan U (2020) Current state of systemic therapies for advanced renal cell carcinoma. Curr Oncol Rep 22:26 32048058 10.1007/s11912-020-0892-1

[CR16] Han Y, Wang Y, Dong X, Sun D, Liu Z, Yue J, Wang H, Li T, Wang C (2023) TISCH2: expanded datasets and new tools for single-cell transcriptome analyses of the tumor microenvironment. Nucleic Acids Res 51:D1425–D1431 36321662 10.1093/nar/gkac959 PMC9825603

[CR17] Horimoto M, Resnick MB, Konkin TA, Routhier J, Wands JR, Baffy G (2004) Expression of uncoupling protein-2 in human colon cancer. Clin Cancer Res 10:6203–6207 15448008 10.1158/1078-0432.CCR-04-0419

[CR18] Hu Y, Wang H, Liu Y (2023) NETosis: sculpting tumor metastasis and immunotherapy. Immunol Rev 321:261–279 10.1111/imr.13277 37712361

[CR19] Huo YX, Huang L, Zhang DF, Yao YG, Fang YR, Zhang C, Luo XJ (2016) Identification of SLC25A37 as a major depressive disorder risk gene. J Psychiatr Res 83:168–175 27643475 10.1016/j.jpsychires.2016.09.011

[CR20] Kaltenmeier C, Yazdani HO, Morder K, Geller DA, Simmons RL, Tohme S (2021) Neutrophil extracellular traps promote T cell exhaustion in the tumor microenvironment. Front Immunol 12:785222 34899751 10.3389/fimmu.2021.785222 PMC8652262

[CR21] Kuai XY, Ji ZY, Zhang HJ (2010) Mitochondrial uncoupling protein 2 expression in colon cancer and its clinical significance. World J Gastroenterol 16:5773–5778 21128330 10.3748/wjg.v16.i45.5773 PMC2997996

[CR22] Lasorsa F, Rutigliano M, Milella M, Ferro M, Pandolfo SD, Crocetto F, Simone S, Gesualdo L, Battaglia M, Ditonno P, Lucarelli G (2023a) Complement system and the kidney: its role in renal diseases, kidney transplantation and renal cell carcinoma. Int J Mol Sci 24:16515 38003705 10.3390/ijms242216515 PMC10671650

[CR23] Lasorsa F, di Meo NA, Rutigliano M, Milella M, Ferro M, Pandolfo SD, Crocetto F, Tataru OS, Autorino R, Battaglia M, Ditonno P, Lucarelli G (2023b) Immune checkpoint inhibitors in renal cell carcinoma: molecular basis and rationale for their use in clinical practice. Biomedicines 11:1071 37189689 10.3390/biomedicines11041071 PMC10136190

[CR24] Lasorsa F, Rutigliano M, Milella M, Ferro M, Pandolfo SD, Crocetto F, Tataru OS, Autorino R, Battaglia M, Ditonno P, Lucarelli G (2023c) Cellular and molecular players in the tumor microenvironment of renal cell carcinoma. J Clin Med 12:3888 37373581 10.3390/jcm12123888 PMC10299533

[CR25] Lewis S, Chen L, Raghuram V, Khundmiri SJ, Chou CL, Yang CR, Knepper MA (2021) “SLC-omics” of the kidney: solute transporters along the nephron. Am J Physiol Cell Physiol 321:C507–C518 34191628 10.1152/ajpcell.00197.2021 PMC8461813

[CR26] Li Q, Wei K, Zhang X, Lv Y, Li M, Zhou C, Su S, Hou D, Hou J (2023) TIMP1 shapes an immunosuppressive microenvironment by regulating anoikis to promote the progression of clear cell renal cell carcinoma. Aging (Albany NY) 15:8908–8929 37688768 10.18632/aging.205005 PMC10522382

[CR27] Lin L, Yee SW, Kim RB, Giacomini KM (2015) SLC transporters as therapeutic targets: emerging opportunities. Nat Rev Drug Discov 14:543–560 26111766 10.1038/nrd4626 PMC4698371

[CR28] Ljungberg B, Albiges L, Abu-Ghanem Y, Bedke J, Capitanio U, Dabestani S, Fernández-Pello S, Giles RH, Hofmann F, Hora M, Klatte T, Kuusk T, Lam TB, Marconi L, Powles T, Tahbaz R, Volpe A, Bex A (2022) European association of urology guidelines on renal cell carcinoma: the 2022 update. Eur Urol 82:399–410 35346519 10.1016/j.eururo.2022.03.006

[CR29] Lucarelli G, Galleggiante V, Rutigliano M, Sanguedolce F, Cagiano S, Bufo P, Lastilla G, Maiorano E, Ribatti D, Giglio A, Serino G, Vavallo A, Bettocchi C, Selvaggi FP, Battaglia M, Ditonno P (2015) Metabolomic profile of glycolysis and the pentose phosphate pathway identifies the central role of glucose-6-phosphate dehydrogenase in clear cell-renal cell carcinoma. Oncotarget 6:13371–13386 25945836 10.18632/oncotarget.3823 PMC4537021

[CR30] Lucarelli G, Rutigliano M, Ferro M, Giglio A, Intini A, Triggiano F, Palazzo S, Gigante M, Castellano G, Ranieri E, Buonerba C, Terracciano D, Sanguedolce F, Napoli A, Maiorano E, Morelli F, Ditonno P, Battaglia M (2017) Activation of the kynurenine pathway predicts poor outcome in patients with clear cell renal cell carcinoma. Urol Oncol 35:461.e15-461.e27 28359744 10.1016/j.urolonc.2017.02.011

[CR31] Lucarelli G, Rutigliano M, Sallustio F, Ribatti D, Giglio A, Lepore Signorile M, Grossi V, Sanese P, Napoli A, Maiorano E, Bianchi C, Perego RA, Ferro M, Ranieri E, Serino G, Bell LN, Ditonno P, Simone C, Battaglia M (2018) Integrated multi-omics characterization reveals a distinctive metabolic signature and the role of NDUFA4L2 in promoting angiogenesis, chemoresistance, and mitochondrial dysfunction in clear cell renal cell carcinoma. Aging (Albany NY) 10:3957–3985 30538212 10.18632/aging.101685 PMC6326659

[CR32] Lucarelli G, Loizzo D, Franzin R, Battaglia S, Ferro M, Cantiello F, Castellano G, Bettocchi C, Ditonno P, Battaglia M (2019) Metabolomic insights into pathophysiological mechanisms and biomarker discovery in clear cell renal cell carcinoma. Expert Rev Mol Diagn 19:397–407 30983433 10.1080/14737159.2019.1607729

[CR33] Lucarelli G, Rutigliano M, Loizzo D, di Meo NA, Lasorsa F, Mastropasqua M, Maiorano E, Bizzoca C, Vincenti L, Battaglia M, Ditonno P (2022) MUC1 tissue expression and its soluble form CA15-3 identify a clear cell renal cell carcinoma with distinct metabolic profile and poor clinical outcome. Int J Mol Sci 23:13968 36430448 10.3390/ijms232213968 PMC9696833

[CR34] Lucarelli G, Netti GS, Rutigliano M, Lasorsa F, Loizzo D, Milella M, Schirinzi A, Fontana A, Di Serio F, Tamma R, Ribatti D, Battaglia M, Ranieri E, Ditonno P (2023) MUC1 expression affects the immunoflogosis in renal cell carcinoma microenvironment through complement system activation and immune infiltrate modulation. Int J Mol Sci 24:4814 36902242 10.3390/ijms24054814 PMC10003656

[CR35] Netti GS, Lucarelli G, Spadaccino F, Castellano G, Gigante M, Divella C, Rocchetti MT, Rascio F, Mancini V, Stallone G, Carrieri G, Gesualdo L, Battaglia M, Ranieri E (2020) PTX3 modulates the immunoflogosis in tumor microenvironment and is a prognostic factor for patients with clear cell renal cell carcinoma. Aging (Albany NY) 12:7585–7602 32345771 10.18632/aging.103169 PMC7202504

[CR36] Papayannopoulos V (2018) Neutrophil extracellular traps in immunity and disease. Nat Rev Immunol 18:134–147 28990587 10.1038/nri.2017.105

[CR37] Peng F, Liao M, Qin R, Zhu S, Peng C, Fu L, Chen Y, Han B (2022) Regulated cell death (RCD) in cancer: key pathways and targeted therapies. Signal Transduct Target Ther 7:286 35963853 10.1038/s41392-022-01110-y PMC9376115

[CR38] Pitt JM, Marabelle A, Eggermont A, Soria JC, Kroemer G, Zitvogel L (2016) Targeting the tumor microenvironment: removing obstruction to anticancer immune responses and immunotherapy. Ann Oncol 27:1482–1492 27069014 10.1093/annonc/mdw168

[CR39] Qi L, Chen F, Wang L, Yang Z, Zhang W, Li Z (2023) Deciphering the role of NETosis-related signatures in the prognosis and immunotherapy of soft-tissue sarcoma using machine learning. Front Pharmacol 14:1217488 37408763 10.3389/fphar.2023.1217488 PMC10318157

[CR40] Ravi P, Mantia C, Su C, Sorenson K, Elhag D, Rathi N, Bakouny Z, Agarwal N, Zakharia Y, Costello BA, McKay RR, Narayan V, Alva A, McGregor BA, Gao X, McDermott DF, Choueiri TK (2020) Evaluation of the safety and efficacy of immunotherapy rechallenge in patients with renal cell carcinoma. JAMA Oncol 6:1606–1610 32469396 10.1001/jamaoncol.2020.2169 PMC7260689

[CR41] Ronchetti L, Boubaker NS, Barba M, Vici P, Gurtner A, Piaggio G (2021) Neutrophil extracellular traps in cancer: not only catching microbes. J Exp Clin Cancer Res 40:231 34261496 10.1186/s13046-021-02036-z PMC8281578

[CR42] Snyder AG, Oberst A (2021) The antisocial network: cross talk between cell death programs in host defense. Annu Rev Immunol 39:77–101 33441019 10.1146/annurev-immunol-112019-072301 PMC8594462

[CR43] Tamma R, Rutigliano M, Lucarelli G, Annese T, Ruggieri S, Cascardi E, Napoli A, Battaglia M, Ribatti D (2019) Microvascular density, macrophages, and mast cells in human clear cell renal carcinoma with and without bevacizumab treatment. Urol Oncol 37:355.e11-355.e19 30738745 10.1016/j.urolonc.2019.01.025

[CR44] Tang D, Kang R, Berghe TV, Vandenabeele P, Kroemer G (2019) The molecular machinery of regulated cell death. Cell Res 29:347–364 30948788 10.1038/s41422-019-0164-5 PMC6796845

[CR45] Vorobjeva NV, Chernyak BV (2020) NETosis: molecular mechanisms, role in physiology and pathology. Biochemistry (mosc) 85:1178–1190 33202203 10.1134/S0006297920100065 PMC7590568

[CR46] Vuong L, Kotecha RR, Voss MH, Hakimi AA (2019) Tumor microenvironment dynamics in clear-cell renal cell carcinoma. Cancer Discov 9:1349–1357 31527133 10.1158/2159-8290.CD-19-0499 PMC6774890

[CR47] Wei K, Zhang X, Yang D (2023) Identification and validation of prognostic and tumor microenvironment characteristics of necroptosis index and BIRC3 in clear cell renal cell carcinoma. PeerJ 11:e16643 38130918 10.7717/peerj.16643 PMC10734432

[CR48] Weide LM, Schedel F, Weishaupt C (2023) Neutrophil extracellular traps correlate with tumor necrosis and size in human malignant melanoma metastases. Biology (Basel) 12:822 37372107 10.3390/biology12060822 PMC10295294

[CR49] Xiang T, Wei Z, Ye C, Liu G (2023) Prognostic impact and immunotherapeutic implications of NETosis-related gene signature in gastric cancer patients. J Cell Mol Med 28:e18087 38146607 10.1111/jcmm.18087 PMC10902305

[CR50] Yin Y, Dai H, Sun X, Xi Z, Zhang J, Pan Y, Huang Y, Ma X, Xia Q, He K (2023) HRG inhibits liver cancer lung metastasis by suppressing neutrophil extracellular trap formation. Clin Transl Med 13:e1283 37254661 10.1002/ctm2.1283 PMC10230156

[CR51] Yu Z, Lu W, Su C, Lv Y, Ye Y, Guo B, Liu D, Yan H, Mi H, Li T, Zhang Q, Cheng J, Mo Z (2021) Single-cell RNA-seq identification of the cellular molecular characteristics of sporadic bilateral clear cell renal cell carcinoma. Front Oncol 11:659251 34168986 10.3389/fonc.2021.659251 PMC8217644

[CR52] Zhang X, Wei X, Wang Y, Wang S, Ji C, Yao L, Song N (2021) Pyroptosis regulators and tumor microenvironment infiltration characterization in clear cell renal cell carcinoma. Front Oncol 11:774279 35070978 10.3389/fonc.2021.774279 PMC8766752

[CR53] Zhang Y, Guo L, Dai Q, Shang B, Xiao T, Di X, Zhang K, Feng L, Shou J, Wang Y (2022) A signature for pan-cancer prognosis based on neutrophil extracellular traps. J Immunother Cancer 10:e004210 35688556 10.1136/jitc-2021-004210 PMC9189842

[CR54] Zhang D, Zhang X, Liu Z, Han T, Zhao K, Xu X, Zhang X, Ren X, Qin C (2023a) An integrative multi-omics analysis based on disulfidptosis-related prognostic signature and distinct subtypes of clear cell renal cell carcinoma. Front Oncol 13:1207068 37427103 10.3389/fonc.2023.1207068 PMC10327293

[CR55] Zhang X, Zhang M, Song L, Wang S, Wei X, Shao W, Song N (2023b) Leveraging diverse cell-death patterns to predict the prognosis, immunotherapy and drug sensitivity of clear cell renal cell carcinoma. Sci Rep 13:20266 37985807 10.1038/s41598-023-46577-z PMC10662159

[CR56] Zhou D, Wu X, Liu X, He S, Ni J, Chen B, Mu D (2023) The pharmacological mechanism of β-elemene in the treatment of esophageal cancer revealed by network pharmacology and experimental verification. Sci Rep 13:12160 37500660 10.1038/s41598-023-38755-w PMC10374640

