# Supplementary material for: Prognostic impact and immunotherapeutic implications of NETosis-related prognostic model in clear cell renal cell carcinoma
Source: J Cancer Res Clin Oncol. 2024 May 27;150(5):278. doi: 10.1007/s00432-024-05761-y (PMC11129999; doi:10.1007/s00432-024-05761-y)
Supplement: Supplementary file 1 — Supplementary file1 (DOCX 123 KB) [file 432_2024_5761_MOESM1_ESM.docx]

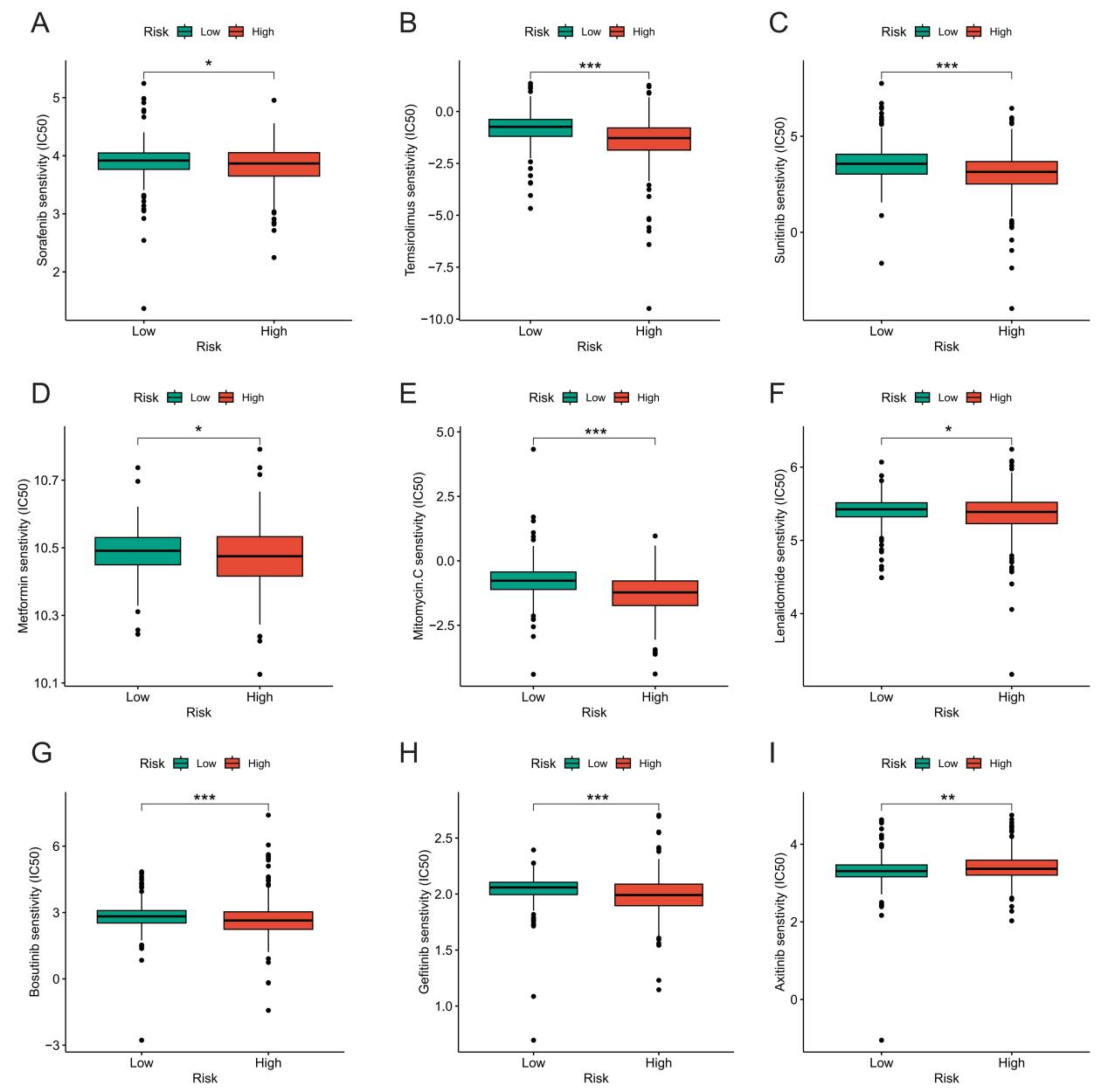


Figure S1. Drug Sensitivity Analysis of NETosis-Related Signature

Sensitivity analysis for (A) Sorafenib, (B) Temsirolimus, (C) Sunitinib, (D) Metformin, (E) Mitomycin C, (F) Lenalidomide, (G) Bosutinib, (H) Gefitinib, and (I) Axitinib between low and high NETosis-related signature groups.
